# Advances in the discovery of exosome inhibitors in cancer

**DOI:** 10.1080/14756366.2020.1754814

**Published:** 2020-06-16

**Authors:** Huarui Zhang, Jun Lu, Jin Liu, Ge Zhang, Aiping Lu

**Affiliations:** a Law Sau Fai Institute for Advancing Translational Medicine in Bone and Joint Diseases (TMBJ), School of Chinese Medicine, Hong Kong Baptist University, Hong Kong, China; b School of Pharmacy, Chengdu University of Traditional Chinese Medicine, Chengdu, China; c Institute of Integrated Bioinfomedicine and Translational Science, Hong Kong Baptist University Shenzhen Research Institute and Continuing Education, Shenzhen, China

**Keywords:** Exosome, extracellular vesicles, cancer, exosome inhibitors

## Abstract

Exosomes are small membrane vesicles released by most eukaryotic cells. They are considered to play an essential role in cell-to-cell communication, and It is also found that they serve as functional mediators in many severe diseases, including progression of various types of cancers. Inhibition of exosome release may slow the progression of some cancers; thus, exosome has been an attractive target for cancer treatment. Over the years, considerable efforts have been made to discover novel, highly potent and excellently selective exosome inhibitors. Most of these inhibitors are derived from synthetic compounds, some of which are currently existed drugs and found to have the potential to inhibit exosome release. In this review, we briefly discussed the development of exosome inhibitors that are currently discovered and provided guidance for the future development of inhibitors.

## Introduction

Exosomes are extracellular vesicles (EVs) that produced in the endosomal compartment of most eukaryotic cells, and have observed increasing attentions over the past decade. The presence of exosomes in extracellular space was identified as early as the late 1980s^1^. Pools of exosomes are packed in the multi-vesicular endosomes (MVEs) and released into the extracellular space after the fusion of MVEs with the plasma membrane^2–4^. The exosomes secreted from cells were initially proposed as cellular waste resulting from cell damage, or by-products of cell homeostasis, and have no significant impact on neighbouring cells. Only recently, these extracellular vesicles are found to play important roles in intercellular communication, they carry a complex cargo of proteins, lipids, and nucleic acids, and then deliver these cargos to the target cells they encounter, which may ultimately reprogramme the recipient cells distal from their release^5–8^. Therefore, as a novel mode of intercellular communication, exosomes may play a major role in many cellular activities, such as signal transduction and immune response^9^. However, exosomes can be released by practically all eukaryotic cells, so their cargos may differ from each other functionally. It is found that exosomes are involved in in various disease processes^10^^,^^11^. In cancer, for example, the tumour-derived exosomes are implicated in promoting tumour progression, angiogenic switch, and immune escape by paracrine subversion of local and distant microenvironments^12^^,^^13^. Their roles in various stages of metastasis, including the induction of migration, invasion and pre-metastatic niche formation, have been well-documented in various human neoplastic diseases.

With the clear understanding of the mechanism of exosomes release, much more efforts have been made to develop therapeutically useful exosome inhibitors as adjunctive therapy for cancer. To date, thousands of papers have been published on the research of exosomes, and a plenty of exosome inhibitors with different skeletons have been discovered. However, only a few reviews on exosome inhibitors have been published. Considering the attractiveness and significance on developing exosome inhibitors for clinical use, in this review, we would, therefore, like to deliver a survey on the recent development of exosome inhibitors.

## Exosome inhibitors

### Exosome inhibitors targeting RAB27A

Ras-related protein RAB27A is a protein that in human which is encoded by the RAB27A gene. The protein encoded by this gene belongs to the small GTPase superfamily, RAB family. This protein is membrane-bound and may be involved in protein transport and small GTPase mediated signal transduction. It is found that RAB proteins could play an important role in exosome production or secretion. The knocking-down of RAB proteins (RAB27A and RAB27B) inhibited exosome secretion without major modifications in the secretion of soluble proteins through the regular secretory pathway. Efforts have been made to discover new compounds that can interfere the function of RAB27A to inhibit exosome release (Table 1). In 2016, Jennifer et al. reported several compounds that can inhibit exosome release in human neutrophils via interfering the interaction between RAB27A and JFC1^14^. They used High-throughput screening (HTS) technique to screen the inhibitors. Two of these compounds Nexinhib4 and Nexinhib20 are proven to be active against the RAB27A-JFC1 complex. These two compounds have the similar structure, both of them have aromatic moiety and nitryl part. But Nexinhib4 was based on thiazole while Nxinhib20 on triazole. It was interesting to note that the activity of Nexinhib20 was 4-fold more active than Nexinhib4. The potential mechanism of Nexinhib20 inhibiting exosome release is that the important residue Tyr122 in RAB27A pockets could mediates pi-pi stacking interactions with Nexinhib20. Thus, by occupying this key residue, Nexinhib20 could interfere the function of RAB27A and inhibit exosome release. Moreover, Nexinhib20 also showed a dose-dependent inhibitory activity on the binding of RAB27A to JFC1 with a calculated IC50 = 2.6 μM.

**Table 1.** RAB27A inhibitors.

Compound	Structure	EC_50_(μM)	Reference
Tipifarnib		1.0	[14,15]
Neticonazole		8.0	[16]
Climbazole		10.0	[16]
Ketoconazole		5.0	[17]
Nexinhib20		2.6	[18]
Nexinhib4		10.0	[18]

Table showing the exosome inhibitors that target RAB27A, their potency and their structures.

**Figure 1.**
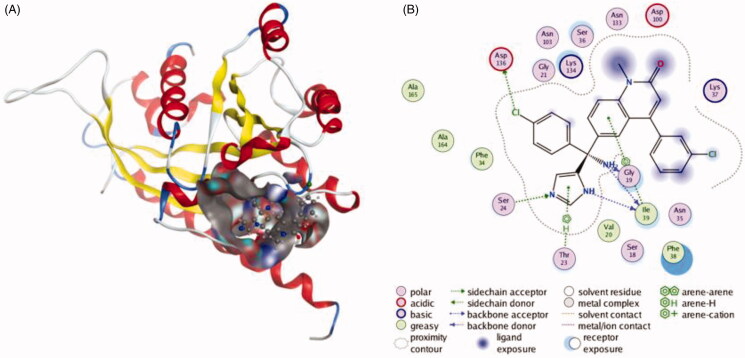
The interactions between Tipifarnib and RAB27A from molecular docking. (A) The pocket is shown in electrostatics representation. (B) The two-dimensional schematic representation of the Rab27a and Tipifarnib complex interactions. Red, yellow, blue and white ribbons: RAB27A. The binding surfaces are identified in grey. The molecular structures of Tipifarnib is displayed by purple ball-and-stick models. Green lines indicate pi-pi stacking interactions, and purple dashed arrows represent sidechain hydrogen bond interactions. Polar and hydrophobic residues are depicted with green and pink circles, respectively.

Amrita et al. screened four currently existed compounds that can selectively inhibit the biogenesis and secretion of exosome to slow down cancer progression^16^. The first one, tipifarnib, is a potent farnesyl transferase inhibitor, it can interfere cell growth and induce cell apoptosis^14^^,^^15^. Its exosome inhibitory effect was evident, data showed that the level of exosome released from PCa cells which exposed to 0.25–1 μM of tipifarnib was significantly decreased. The underpinning mechanism of tipifarnib is inhibiting the expression of RAB27A, Alix and nSMase2^19^. Additionally, the inhibitory effect of tipifarnib may be selective for cancer cells, because it only affects exosome release in C4-2B and PC-3 cells but not in the RWPE-1 cells (Human Prostate Epithelial cells). This property is crucial for the clinical use of exosome inhibitors. The interactions between tipifarnib and RAB27A are shown in Figure 1. The inhibitory effects of neticonazole and climbazole were also measured, both of them (20 µM) could significantly decrease exosome secretion by downregulating the level of Alix and Rab27a, additionally neticonazole also decreased nSMase2 levels. This team also screened a compound that has currently been approved by the United States for PCa patients, ketoconazole, could inhibit exosome release^17^. Ketoconazole (5 μM) decreased the level of exosome produced by C4-2B cells and PC-3 cells, and increasing concentrations (0–5 μM) led to a robust dose-dependent decrease in RAB27A, Alix and nSMase2 in both C4-2B and PC-3 cells, but not in the RWPE-1 cells. Interestingly, although the structures of these four compounds are different from each other, they all have the same parts, diazole and aromatic moiety, which suggests the importance of both to the structure of the drugs. Furthermore, the activities of these compounds are quite different, the efficacy of tipifarnib is much more active than the other three compounds.

### Exosome inhibitors targeting sphingomyelinase

**Figure 2.**
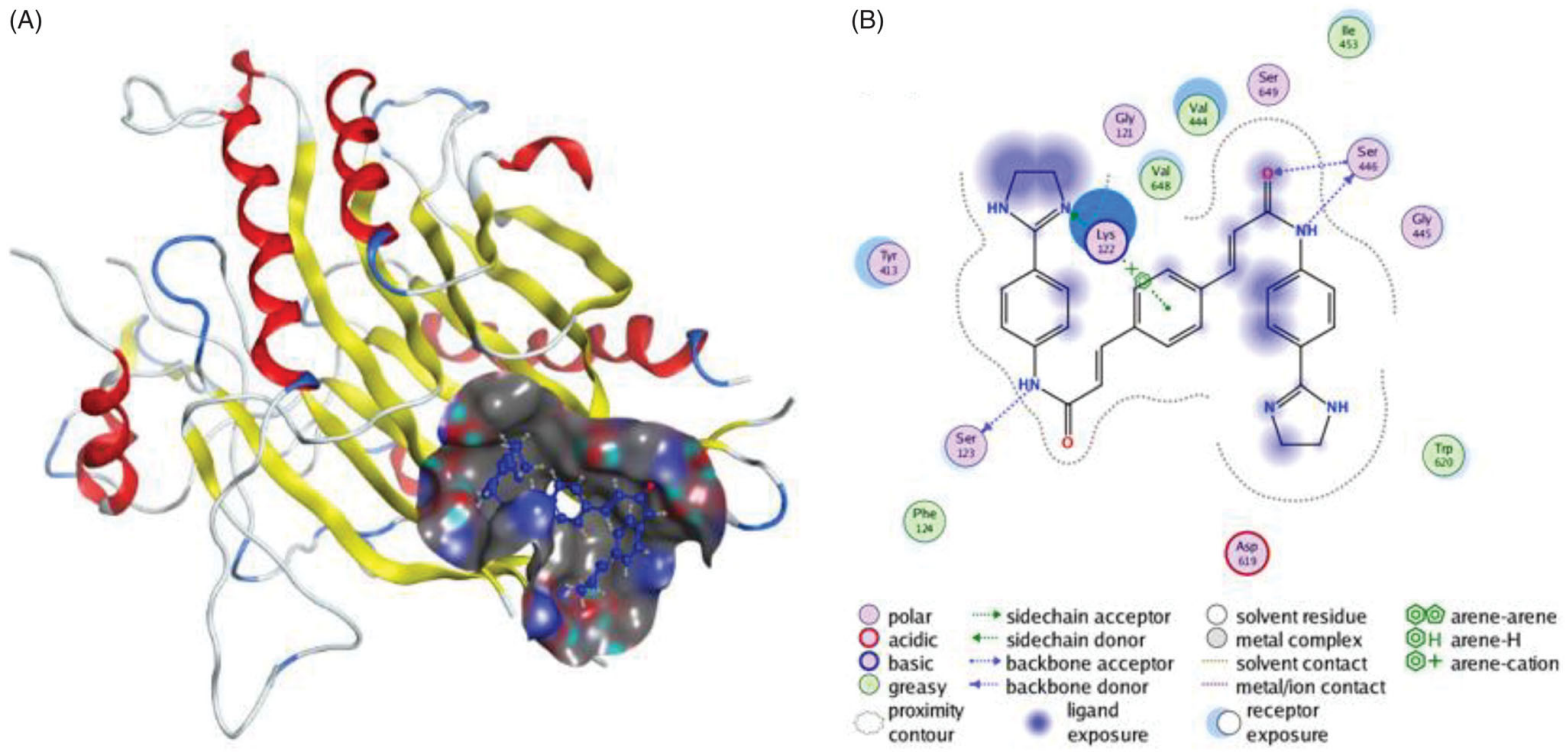
The interactions between GW4869 and nSMase from molecular docking. (A) The pocket is shown in electrostatics representation. (B) The two-dimensional schematic representation of the nSMase and GW4869 complex interactions. Red, yellow, blue and white ribbons: nSMase. The binding surfaces are identified in grey. The molecular structures of GW4869 is displayed by purple ball-and-stick models. Green lines indicate pi-pi stacking interactions, and purple dashed arrows represent sidechain hydrogen bond interactions. Polar and hydrophobic residues are depicted with green and pink circles, respectively.

Sphingomyelinase (SMase) is a hydrolase enzyme that is involved in sphingolipid metabolism reactions. SMase family contains alkaline, neutral, and acidic SMase depending on the pH in which their enzymatic activity is optimal. They are responsible for breaking sphingomyelin (SM) down into phosphocholine and ceramide^20^. Researchers have found that ceramide could regulate exosome production, and the inhibition of neutral sphingomyelinase (nSMase) could downregulate the level of ceramide^21^^,^^22^. As a result, inhibiting nSMase could reduce the amount of released exosome^23^^,^^24^. Therefore, nSMase is a potential therapeutic target for inhibiting exosome release. Efforts have been made to develop nSMase inhibitors as exosome inhibiting agents (Table 2). The first nSMase inhibitor that has been used to inhibit the production of exosome is GW4869^22^^,^^25–30^. This compound has been successfully used to block the secretion of exosomes from MCF-7 cells, lung epithelial cells and RAW264.7 macrophages^29^^,^^39^^,^^40^. Around twenty-two percent reduction of exosome was detected when treated RAW264.7 macrophages with 10 μM GW4869, and the inhibitory effect was enhanced when the concentration was raised to 20 μM. Additionally, the level of ceramide was significantly reduced. The interactions between GW4869 and nSMase are shown in Figure 2. Another neutral sphingomyelinase inhibitor, Manumycin A (MA), also has the ability to block the secretion of exosomes^27^^,^^28^. According to exosome quantification analysis, MA (250 nM) significantly suppressed exosome secretion in C4-2B, 22Rv1, and PC-3 cells by about 55%, as compared to the controls. More importantly, the exosome inhibitory effect of MA did not appear in the normal RWPE-1 cells which means that MA could selectively inhibit the exosome released from cancer cells. This phenomenon suggests that MA could be a potential exosome inhibiting agent in the future. A natural product, spiroepoxide, also has been reported to block exosome release^29^^,^^30^. At 5 μM, spiroepoxide can inhibit exosome release by 20% which is as effective as GW4869 at the same concentration. However, the structure of this compound is totally different from GW4869. GW4869 has three benzene rings and two diazole groups while spiroepoxide only has one benzene ring and one ternary ring.

**Table 2.** nSMase inhibitors.

Compound	Structure	EC_50_(μM)	Reference
GW4869		1.0	[22,25,26]
Manumycin A		0.25	[27,28]
Spiroepoxide		2.0	[29,30]
Cambinol		5.0	[31]
Scyphostatin		N/A	[32,33]
DPTIP		2.0	[34]

Table showing the exosome inhibitors that target nSMase, their potency and their structures.

Through screening, Camilo Rojas et al. discovered a newly identified potent brain penetrant neutral sphingomyelinase 2 inhibitor to cure brain injury, which name was 2,6-Dimethoxy-4–(5-Phenyl4-Thiophen-2-yl-1H-Imidazol-2-yl)-Phenol (DPTIP)^34^. DPTIP was described as the most potent nSMase2 inhibitor that has been reported so far. The IC_50_ of DPTIP is 30 nM which is much more potent than the prototype inhibitors GW4869 (1 µM) and cambinol (5 µM)^25^^,^^31^. Moreover, DPTIP is also the first nSMase2 inhibitor described with nanomolar potency. DPTIP’s exosome inhibitory effect was found related to its hydroxyl group, since the des-hydroxyl analogue of DPTIP showed no inhibition effect. Additionally, the inhibitory effect of DPTIP is unique to nSMase2, it does not inhibit members of two related enzyme families including alkaline phosphatase or acid sphingomyelinase, a phosphodiesterase closely related to nSMase2. It is also found that DPTIP blocks EV secretion in a dose dependent manner (0.03–30 µM), and at 30 µM, this compound could decrease exosome release by 50% in astrocytes. Therefore, the exosome inhibitory effect of DPTIP is significant, and it might be a potential agent for synergistic treatment of cancer in the future.

There are some other nSMase inhibitors with high activity. Cambinol, for example, is a novel uncompetitive nSMase inhibitor, its inhibitory activity for nSMase2 (IC50 = 6 μM) was about 10-fold more potent than for its previously known target, silence information regulator 1 and 2 (SIRT1/2)^38^. Scyphostatin, also a well-known nSMase inhibitor, could inhibit nSMase3 in a dose-dependent manner (0–5 μM), and nSMase2 was equally sensitive to inhibition by scyphostatin^32^^,^^33^^,^^41^^,^^42^. However, the exosome inhibitory effects of these two compounds are yet to be verified.

### Other inhibitors

Except for the two kinds of exosome inhibitors above, there are other strategies to inhibit exosome release with different mechanisms (Table 3). In 2009, Parolini et al. reported that the microenvironmental pH of tumour cells is an essential factor for exosome traffic^53^. At low pH condition, the level of released exosomes increased in melanoma cells, and this low pH condition did not affect cell viability. Moreover, the exosome uptake also increased in cells cultured in an acidic condition. According to another research, exosome release can be reduced by alkalising the tumour cell microenvironment^54^. Data showed that as the pH of the microenvironment increased the number of released exosomes progressively decreased in SKBR3, Me30966 and LNCaP cells (exosome released from cells cultured at pH 7.4 conditions were about 20-fold lower than at pH 6.5 conditions). In cancer cells, proton transporter V-ATPases plays a kay role in maintaining an alkaline intracellular pH and an acidic extracellular pH^55^^,^^56^. Therefore, the inhibition of V-ATPases might be a new strategy to block exosome release. It is shown that proton pump inhibitors (PPIs) which have been largely used for treating peptic diseases because of their anti-acidic properties could be used in cancer therapy, and because of PPIs’ V-ATPases inhibiting effect, they can be used to block exosome release as well^53^^,^^56–59^. In 2013, Federici et al. showed that treating tumour cells with PPI induced reduction of released exosome^43^. The *in vitro* study revealed that 50 μM of Lansoprazole (PPI) pre-treatment for one day on human melanoma cells led to a marked reduction in the level of released exosomes compared to the control. Furthermore, the *in vivo* study also indicated that PPI markedly reduced the level of plasmatic exosomes released by human tumour cells. This team also reported other commonly used PPIs that could be used to inhibit the acidification of the tumour microenvironment. In 2004, they found that the pre-treatment of PPIs omeprazole, esomeprazole, or pantoprazole could reverse the resistance of human tumour cells to cytotoxic drugs^44^^,^^56^. It is also discovered that the ability of tumour cells (melanomas, adenocarcinomas, and lymphoma cell lines) to acidify the extracellular medium were impaired after the treatment of omeprazole, and the activity of V-H^+^-ATPase was also inhibited. Similar results were obtained with esomeprazole and pantoprazole. The evidence suggests that these three PPIs could also be used to block exosome release. Another proton exchanger carbonic anhydrase IX (CA IX) which overexpressed in many types of cancers, also played an essential role in tumour pH regulation^60^^,^^61^. Study showed that exosomes purified from the plasma of prostate cancer patients express a high level of CA IX than normal tissue and the concentration of CA IX at the plasma membrane suggests an increased activity of the endosomal compartment, in turn, leading to exosome formation and extracellular release^62^. All these results indicated that CA IX could be a new therapeutic target to interfere exosome release in hypoxic tumours. Moreover, currently, there is already one CA IX inhibitor (SLC-0111) in Phase Ib/II clinical trials for the treatment of hypoxic, metastatic tumors^45^^,^^46^^,^^63–67^. PPIs and CA IX inhibitors are both inhibiting exosome release by regulating the pH of tumour microenvironment. This strategy is novel and efficient, and provides insight for the future development of exosome inhibitors.

**Table 3.** Other inhibitors.

Compound	Structure	EC_50_(μM)	Reference
Lansoprazole		N/A	[43]
Omeprazole		10	[44]
Esomeprazole		70	[44]
Pantoprazole		N/A	[44]
SLC-0111		0.2	[45,46]
Cannabidiol		5.0	[47,48]
Ketotifen		10.0	[49,50]
Simvastatin		1.0	[51]
Sulphisoxazole		50.0	[52]

Table showing the exosome inhibitors that target other proteins, their potency and their structures.

Cannabidiol (CBD), which is a phytocannabinoid derived from Cannabis sativa, has anti-inflammatory, analgesic, antineoplastic and chemo-preventive activities, and has currently been used as a anxiolytic^47^^,^^48^^,^^68^^,^^69^. Recently, it is found that CBD can block exosome and microvesicle (EMV) release^70^^,^^71^. Research data indicated that CBD can block exosome release by 50% at 5 µM and it can selectively inhibit the release exosomes from cancer cell lines (prostate cancer PC3, hepatocellular carcinoma HEPG2 and breast adenocarcinoma MDA-MB-231). Because of its selectivity, it is a very promising agent without many side effects. The underlying mechanism of CBD inhibiting exosome release is found related to its CD63 interfering effect, because the expression of CD63 significantly decreased in all three cell lines after 1 h CBD treatment. In 2018, Khan et al. has reported that Ketotifen (antihistamine), a store-operated calcium channel blocking agent which is used as mast cell stabiliser, has the ability to block exosome release^49^^,^^50^. At 10 µM of ketotifen, the exosome released by HeLa, MCF7 and BT549 cells decreased by 70%, 45% and 30%, respectively. Surprisingly, the effect of ketotifen on exosome increases the sensitivity of cancer cells to doxorubicin and also suppresses the progression of cancer cells^49^^,^^72^. As ketotifen was reported to block calcium influx into cells, and It is shown that exosome release was regulated by calcium-dependent mechanisms, and inhibitors of calcium entry into the cells reduce exosome release^73^^,^^74^. Therefore, the mechanism of ketotifen inhibiting exosome release might due to its calcium channel blocking effect. Furthermore, as interfering the calcium channel could inhibit exosome release, so apply calcium channel blocking agent might be a new strategy to inhibit exosome release.

**Figure 3.**
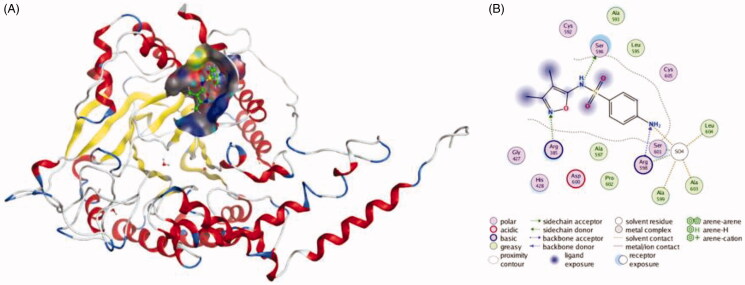
The interactions between Sulphisoxazole and endothelin receptor A from molecular docking. (A) The pocket is shown in electrostatics representation. (B) The two-dimensional schematic representation of the endothelin receptor A and Sulphisoxazole complex interactions. Red, yellow, blue and white ribbons: endothelin receptor A. The binding surfaces are identified in grey. The molecular structures of Sulphisoxazole is displayed by purple ball-and-stick models. Green lines indicate pi-pi stacking interactions, and purple dashed arrows represent sidechain hydrogen bond interactions. Polar and hydrophobic residues are depicted with green and pink circles, respectively.

Simvastatin, which is often used to decrease elevated lipid levels and the risk of heart problems in those at high risk, also exhibited the ability to inhibit the secretion of exosome^51^. Data showed that epithelial cells and monocytes treated with different concentrations of simvastatin for 24 h exhibited a significant reduction in the level of secreted exosomes, and a significant reduction of about 40% was noted at the 0.3 µM dose of simvastatin, so its exosome inhibitory effect was significant. Besides, the levels of exosome-associated proteins were detected, and it is shown that levels of Alix, CD63 and CD81 significantly decreased. Thus, reduction in the levels of exosome synthesising proteins may partly explain the mechanisms of simvastatin-mediated reduction in exosome secretion. Eun-Ju Im et al. found that sulphisoxazole (SFX), a sulphonamide antibacterial, has the ability to block exosome release by targeting the endothelin receptor A^74^. SFX is an orally administered FDA-approved drug without cytotoxicity at effective doses, and this drug was first known to be a competitive inhibitor of the enzyme dihydropteroate synthetase by preventing the condensation of pteridine with *p*-aminobenzoic acid, a substrate of the enzyme in prokaryotic systems^75^. The exosome inhibitory effect of SFX is conspicuous. At 50 µM, the amount of exosome released from MCF7 and MDA-MB231 cells decreased about 50%. In addition, the expression of late endosomal proteins, such as RAB7 and CD63, and RAB27a decreased in the presence of SFX. Moreover, it is found that SFX significantly suppressed the levels of a transcription factor MITF (Microphthalmia-associated transcription factor), which can increase the expression of late endosomal proteins^76^. Hence, the down-regulation of MITF might partly explain the mechanism of SFX inhibiting exosome release. The interactions between Sulphisoxazole and endothelin receptor A are shown in Figure 3. By comparison, all these compounds are different kinds of agents, they are used to treat different diseases, but they have one thing in common, which is that they can inhibit exosome release. Interestingly, the mechanisms of these compounds blocking the secretion of exosome varies from each other. This suggests that during the development of exosomal inhibitors, effective agents can be discovered by aiming at different stages of exosome biogenesis and secretion.

## Conclusions

Exosomes are constantly released by most eukaryotic cells, they were long considered as byproducts of membrane biosynthesis and shedding^77^^,^^78^. Only currently, their role in cell-to-cell communications and multiple important biological functions of exosome have been discovered^79^. The biogenesis/secretion and “cargo” contents of exosomes are more regulated during cancer progression, and the exosome released from normal cells and cancer cells are quantitatively and qualitatively different^80–85^. Current study has shown that cancer progression occurs due to continuous information exchange between the tumour cells and their stromal microenvironment^86^. Exosomes can both induce and facilitate a pro-tumoral microenvironment for the initiation of tumorigenesis, and they can also regulate the immune response to prime tumour progression and survival by promotion of angiogenesis, metastasis and drug resistance^10^^,^^87–89^. Clinical studies showed that exosomes are potential biomarkers in tumour diagnostics, and the increased levels of exosomes might represent a hallmark of malignant cancers and could be used as an indicator of clinical status^90^. For instance, researchers found that the level of exosome was decreased after the treatment of imatinib on gastrointestinal stromal tumor^91^. In colon cancer patients, a novel EV-associated cancer biomarker HSP60 was significantly decreased after the surgical removal of the tumor^92^. Therefore, there are a great deal of proofs on the clinical use of exosomes, and these results indicated that exosomes are used as disease biomarkers. Therefore, the urge for developing agents that may selectively target exosomes from cancer cells is significant. Moreover, as the exosome-associated proteins and mechanisms have been studied for years, plenty of agents have been developed to reduce release of exosomes from cancer cells or reduce their uptake by the recipient cells^93^^,^^94^. These compounds target different proteins and different stages of exosome biogenesis process, they can be RAB27A inhibitors, nSMase inhibitors, PPIs and calcium channel blocking agents, so the mechanisms of these inhibitors are different. Additionally, with further research, some compounds which are used clinically to cure other diseases were found to have the ability to inhibit exosome release, like tipifarnib, ketoconazole, cambinol and simvastatin. It is worth noting that some of these compounds, like tipifarnib, ketoconazole, MA and CBD, only affect the exosome released from tumour cells and have no effect on the exosome secreted by normal cells, which is vital for drug development. As the important roles of exosomes are widely understood, in the future, exosome inhibitors will become increasingly important as synergistic agents for cancer treatment. Hence, developing strategies that target exosome-mediating physiological and pathological communications between cells will have significant therapeutic potential in cancers including other diseases. In a word, it is crucial to develop pharmacological agents that can selectively reduce exosome release, uptake without many side effects, and the current research can provide a reference for future drug development.
